# Antibody engineering improves neutralization activity against K417 spike mutant SARS-CoV-2 variants

**DOI:** 10.1186/s13578-022-00794-7

**Published:** 2022-05-17

**Authors:** Lili Li, Meiling Gao, Peng Jiao, Shulong Zu, Yong-qiang Deng, Dingyi Wan, Yang Cao, Jing Duan, Saba R Aliyari, Jie Li, Yueyue Shi, Zihe Rao, Cheng-feng Qin, Yu Guo, Genhong Cheng, Heng Yang

**Affiliations:** 1 Institute of Systems Medicine, Chinese Academy of Medical Science & Peking Union College, Beijing, 100005 China; 2 Suzhou Institute of Systems Medicine, Suzhou, 215123 China; 3 State Key Laboratory of Medicinal Chemical Biology and College of Life Sciences, Nankai University, Tianjin, 300071 China; 4 Department of Virology, State Key Laboratory of Pathogen and Biosecurity, Beijing Institute of Microbiology and Epidemiology, AMMS, Beijing, 100071 China; 5 AtaGenix Laboratories (Wuhan) Co., Ltd, Wuhan, 430075 China; 6 Center of Growth, Metabolism and Aging, Key Laboratory of Bio-Resource and Eco-Environment of Ministry of Education, College of Life Sciences, Sichuan University, Chengdu, 610065 China; 7 Department of Microbiology, Immunology & Molecular Genetics, University of California, Los Angeles, CA 90095 USA; 8 Department of Laboratory Medicine, Taihe Hospital, Hubei University of Medicine, Shiyan, 442000 China; 9 Guangzhou Laboratory, B1, Standard Property Unite 4, Guangzhou international bio-island, Guangzhou, 510320 China

**Keywords:** SARS-CoV-2 variants, COVID-19, Neutralizing antibody, Phage display library, Antibody engineering

## Abstract

**Background:**

Neutralizing antibodies are approved drugs to treat coronavirus disease-2019 (COVID-19) patients, yet mutations in severe acute respiratory syndrome coronavirus (SARS-CoV-2) variants may reduce the antibody neutralizing activity. New monoclonal antibodies (mAbs) and antibody remolding strategies are recalled in the battle with COVID-19 epidemic.

**Results:**

We identified multiple mAbs from antibody phage display library made from COVID-19 patients and further characterized the R3P1-E4 clone, which effectively suppressed SARS-CoV-2 infection and rescued the lethal phenotype in mice infected with SARS-CoV-2. Crystal structural analysis not only explained why R3P1-E4 had selectively reduced binding and neutralizing activity to SARS-CoV-2 variants carrying K417 mutations, but also allowed us to engineer mutant antibodies with improved neutralizing activity against these variants. Thus, we screened out R3P1-E4 mAb which inhibits SARS-CoV-2 and related mutations in vitro and in vivo. Antibody engineering improved neutralizing activity of R3P1-E4 against K417 mutations.

**Conclusion:**

Our studies have outlined a strategy to identify and engineer neutralizing antibodies against SARS-CoV-2 variants.

**Supplementary Information:**

The online version contains supplementary material available at 10.1186/s13578-022-00794-7.

## Background

Coronavirus disease-2019 (COVID-19), caused by the emerging severe acute respiratory syndrome coronavirus (SARS-CoV-2), is a worldwide pandemic infectious disease. New SARS-CoV-2 variants raised concerns on viral escape from current antibody therapies and vaccine protection. Recently identified variants of SARS-CoV-2 (B.1.1.7 in the United Kingdom, B.1.351 in South Africa, P.1 in Brazil and B.1.617 in India) not only rapidly displacing local SARS-CoV-2 strain, but also carry N-terminal domain (NTD) and receptor binding domain (RBD) mutations that are critical for interaction with neutralizing antibodies [1–4]. B.1.1.7, also known as 501Y.V1, is associated with mutations in its spike (S) protein, including ΔH69/V70 and ΔY144 in NTD, N501Y in RBD, and P681H near the furin cleavage site. B.1.351 (also known as 501Y.V2) and P.1 (also known as 501Y.V3) each have three mutations in common with the RBD (K417N/T, E484K and N501Y) and various mutations in the NTD domain, such as L18F, D80A and D215G in B.1.351 and L18F, T20N, P26S, D138Y, and R190S in P.1. Besides N501Y mutation shared with B.1.1.7, B.1.351 and P.1, B.1.617 is associated with unique L452R, E484Q and A570D mutations in the RBD domain [5–7]. All the above four prevalent variants share D614G mutation, a variant with a single substitute rapidly became the dominant strain in the world and further evolved to given several variants of concern (VOCs) [8–10]. All these mutations show varying impact on antibody therapies and vaccine protection [11–15].

Passive antibodies administered are one of the most promising therapeutic and prophylactic anti-SARS-CoV-2 agents. To date, the most potent monoclonal antibodies (mAbs) isolated from infected and vaccinated individuals were often dominant by those targeting RBD while many isolated NTD mAbs failed to reach 100% potency in neutralizing activity [16–20]. All mAbs authorized or in development are directed to the RBD, which interacts with the target receptor angiotensin converting enzyme 2 (ACE2) [2, 21, 22]. Thus, mutations located within or nearby RBD domain will affect the conformation and affinity between RBD and ACE2, resulting in change of SARS-CoV-2 host-cell interaction and susceptibility to mAbs-mediated neutralization [7, 13, 23]. These results suggest greater impact of SARS-CoV-2 variants on RBD antibodies.

Recently, multiple reports indicate the impacts of SARS-CoV-2 variants on mAbs. Most mAbs were disrupted by the K417N/T mutation and/or E484K mutation possessed by B.1.351 and P.1 variants within the RBD domain. B.1.351 and P.1 are reported to resistant to neutralization by many anti-RBD and anti-NTD antibodies, including CB6, REGN10933, Bamlanivimab and Casirivimab which are already approved for emergence use authorization (EUA) [14, 24, 25]. B.1.351 and B.1.1.7 variants are also observed to show decreased neutralizing activity on RBD mAbs from vaccine-elicited individuals and COVID-19 convalescents [6, 26]. Antibody resistance of SARS-CoV-2 variants highlighted the importance of understanding the mechanisms responsible for the escape of antibody neutralization by different SARS-CoV-2 variants, and the urgent need for novel antibody development strategy to overcome the antibody resistance problem caused by the dominant mutations associated with SARS-CoV-2 VOCs.

In this work, we have identified multiple human mAbs through scFv phage display library derived from COVID-19 patients. We have further characterized one of them, R3P1-E4, and demonstrated its efficacy in suppressing SARS-CoV-2 in cultured cells and in mice. Furthermore, our crystal structural analysis on the R3P1-E4 and RBD complex have not only explained the mechanism responsible for the reduced neutralization activity of R3P1-E4 against B.1.351. and P.1 variants, but also engineered R3P1-E4 mutant antibodies with improved neutralization activity against these VOCs.

## Results

### Screening for antibodies that bind to SARS-CoV-2 S protein RBD domain

**Fig. 1:**
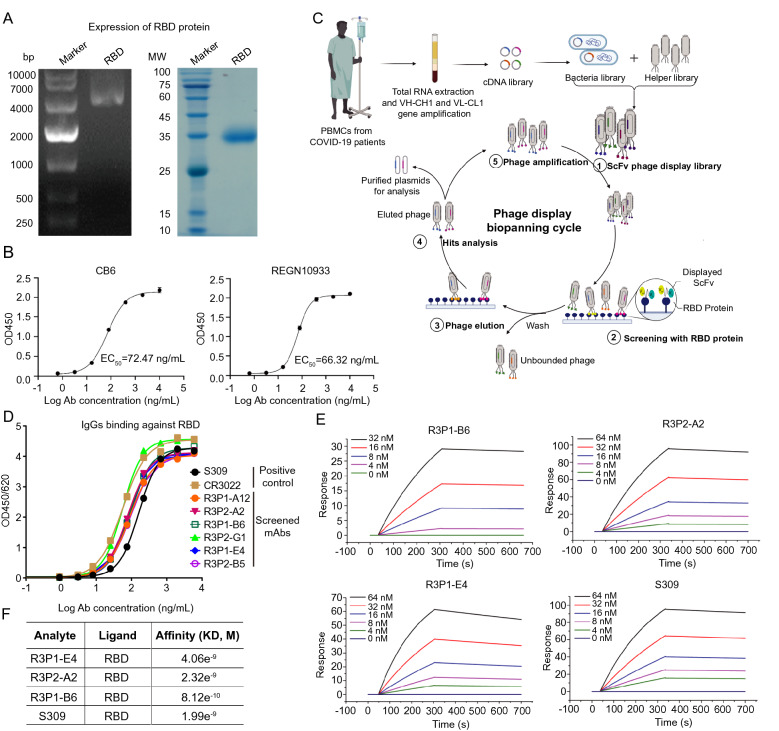
Screening for antibodies that bind to SARS-CoV-2 S protein RBD domain. **A** DNA electrophoresis (left panel) and SDS-PAGE (right panel) were performed to confirm the expression and purification of SARS-CoV-2 S RBD protein. **B** The binding of purified S RBD protein to CB6 and REGN10933 mAbs was examined by ELISA assay. **C** Sketch map of COVID-19 patients’ scFv phage display library construction and mAbs screening. Briefly, VH and VL genes amplified and cloned from COVID-19 patients’ PBMC were inserted into T7 bacteriophage vector and packaged into phage particles displaying the scFv on their surfaces. The phage library was mixed with RBD protein that binds to their cognate epitopes. Bounded phage were eluted by immunoprecipitation with protein A/G coated magnetic beads. Last, PCR amplification and Illumina sequencing from the DNA of the bound phage were performed to reveal the sequence of scFv. **D** The binding of screened six mAbs to RBD protein was examined by ELISA assay. S309 and CR3022 Abs were used as positive control. **E** Sensorgrams of the binding of R3P1-B6, R3P2-A2, R3P1-E4 and S309 mAbs to RBD protein. The Ab concentrations were used as indicated. **F** The K_D_ values in (**E**)

We firstly constructed and expressed the RBD domain of SARS-CoV-2 S protein, and verified the construction and expression of RBD domain by DNA electrophoresis and SDS-PAGE (Fig. 1A). ELISA assay confirmed that the RBD domain effectively binds with two previously published human mAbs specifically binding to the RBD domain of SARS-CoV-2 S protein: CB6 and REGN10933 (Fig. 1B). Then, the expressed and purified RBD protein was used as bait to screen mAbs from scFv phage display library derived from COVID-19 patients’ peripheral blood mononuclear cells (PBMC) as described previously [27] (Fig. 1C). Through this scFv phage display technique, we have identified six human mAbs, R3P1-A12, R3P2-A2, R3P1-E4, R3P2-B5, R3P1-B6 and R3P2-G1, which were subsequently cloned into human IgG backbone, expressed in Chinese hamster ovary (*CHO*) cells and purified. The binding of these mAbs with RBD protein were verified by ELISA with two known SARS-CoV-2 neutralizing antibodies, S309 and CR3022, as positive controls (Fig. 1D). The results showed that the six mAbs bound well with the RBD protein, all of which had similar affinities as S309 and CR3022. We have further conducted the surface plasmon resonance (SPR) assay and found that the binding affinities of R3P1-B6, R3P2-A2 and R3P1-E4 with RBD were 4.06, 2.32 and 0.82 nM, which were similar to 1.99 nM affinity for the S309 antibody (Fig. 1E, F). These results indicate that we have identified six human mAbs with high binding affinity to the RBD domain of SARS-CoV-2 S protein from the scFv phage display library generated from COVID-19 patients.

### Sequencing and functional characterization of anti-SARS-CoV-2 RBD mAbs

**Fig. 2:**
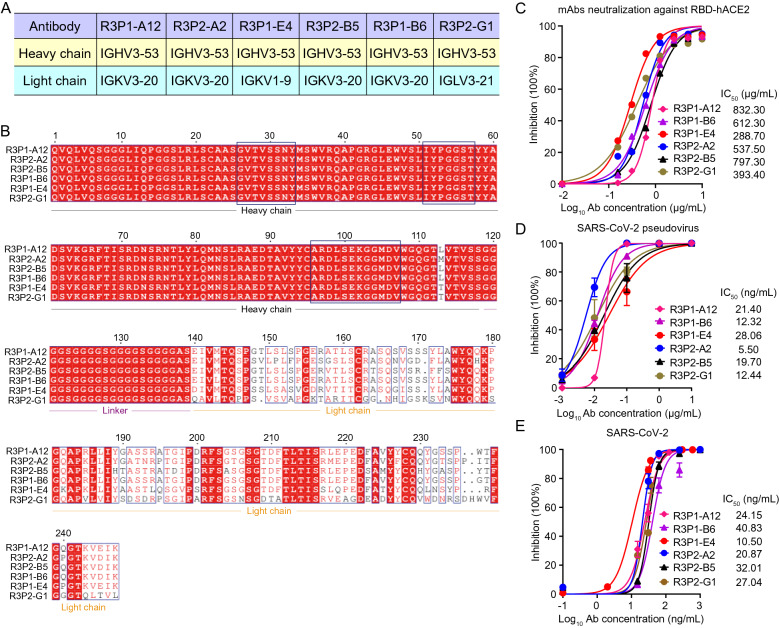
R3P1-E4 suppresses SARS-CoV-2 pseudovirus and authentic virus infection in vitro*.* **A** Germline usage comparison of heavy chain and light chain of R3P1-A12, R3P2-A2, R3P1-E4, R3P2-B5, R3P1-B6 and R3P2-G1 mAbs. **B** Alignment of the heavy chain and light chain variable domain sequence of R3P1-A12, R3P2-A2, R3P1-E4, R3P2-B5, R3P1-B6 and R3P2-G1 mAbs. The sequences in the box are variable region of mAbs. **C** Antibody competition with hACE2 receptor assay were performed to test the abilities of screened mAbs to competitively bind with RBD protein against hACE2. **D** The abilities of screened mAbs to neutralize SARS-CoV-2 pseudovirus were examined by pseudovirus neutralization assay. **E** The abilities of screened mAbs to neutralize SARS-CoV-2 authentic virus were examined by authentic virus neutralization assay

We sequenced the scFv regions of the above six human mAbs and found in surprise that the R3P1-A12, R3P2-A2, R3P1-E4, R3P2-B5, R3P1-B6 and R3P2-G1 mAbs all shared almost identical heavy chain sequences except one amino acid variations. This heavy chain contained the immunoglobulin heavy variable 3–53 (IGHV3-53) segment of the human immunoglobulin gene (Fig. 2A, B). Interestingly, several previously reported mAbs including P4A1, CC12.1, CC12.3 and B38, which all known bind to the receptor binding motif (RBM) region of SARS-CoV-2, also shared the same IGHV3-53 gene segment (Additional file 1: Fig. S1), suggesting this IGHV gene segment was selected in COVID-19 patients. Although the heavy and light chains were randomly paired in the phage display library, IGKV3-20 were selected for R3P1-A12, R3P2-A2, R3P2-B5 and R3P1-B6 mAbs, while IGKV1-9 and IGLV3-21 were selected for R3P1-E4 and R3P2-G1, respectively (Fig. 2A, B). Interestingly, both P4A1 and CC12.3 mAbs used IGKV3-20 and CC12.1 and B38 used IGKV1-9 (Additional file 1: Fig. S1), suggesting that these light chain fragments were selected in COVID-19 patients.

To test the potential anti-viral function of the six mAbs on SARS-CoV-2, we first measured their abilities to neutralize the binding between RBD and hACE2. As the increasing concentrations of these six mAbs added, the binding activities between RBD and hACE2 were gradually decreased (Fig. 2C), suggesting that the six mAbs possessed the abilities to compete with hACE2 on RBD binding. We also performed antibody neutralization assay with SARS-CoV-2 pseudovirus, which was engineered HIV-based pseudoviruses with full length S protein expressed on the surface of viral particles. The results showed that all the six mAbs effectively suppressed SARS-CoV-2 pseudovirus (Fig. 2D). In order to measure the efficacy of individual mAbs in inhibiting the replication of authentic SARS-CoV-2, we pre-incubated live SARS-CoV-2 viruses with six different concentrations of the six mAbs ranging from 0.1 to 1000 ng/ml before infecting vero cells and measured the levels of viral genome RNA at 24 h post infection. The results showed that all the six mAbs inhibited SARS-CoV-2 authentic virus, and R3P1-E4 showed the smallest IC_50_ values among these six mAbs (Fig. 2E). These results suggest that R3P1-E4 is a potential effective antibody for SARS-CoV-2 suppression.

### R3P1-E4 suppresses SARS-CoV-2 infection in vivo

**Fig. 3:**
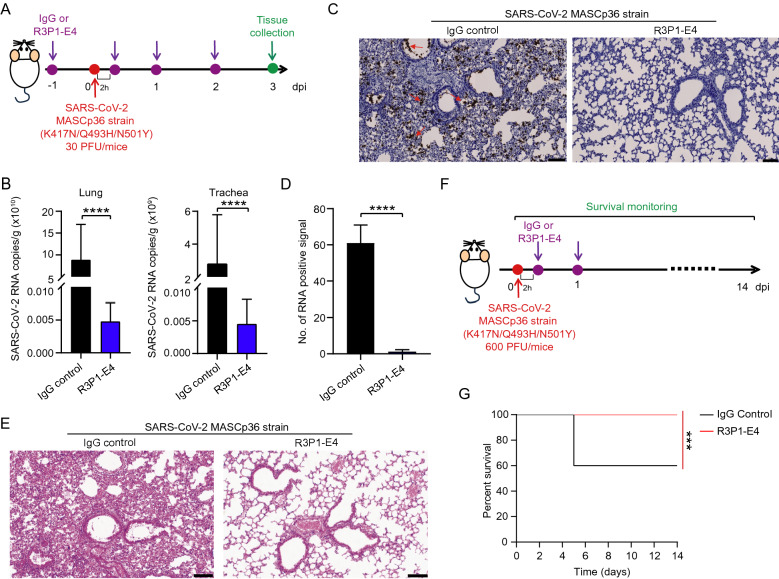
R3P1-E4 suppresses SARS-CoV-2 infection in vivo. **A–C** The in vivo antiviral effect of R3P1-E4 against SARS-CoV-2. 8-month-old male mice were administrated intraperitoneally with R3P1-E4 Ab (25 mg/kg) (n = 5) or IgG control (n = 5) 24 h before and 24, 48 and 72 h after intranasal challenge with the SARS-CoV-2 mouse adapted strain MASCp36 (30 PFU/mouse) (**A**). Lung and trachea viral loads were tested by qRT-PCR (**B**) and RNA scope (**C**) at 3 dpi. **D** The quantification of (**C**). **E** The lung damage caused by SARS-CoV-2 infection was examined by H&E staining. Scale bar: 100 μm. **F-G** The in vivo antiviral effect of R3P1-E4 against SARS-CoV-2. 8-month-old male mice were administrated intraperitoneally with R3P1-E4 Ab (25 mg/kg) (n = 5) or IgG control (n = 5) at 2 h and 24 h after infection with SARS-CoV-2 MASCp36 strain (600 PFU/mouse) (**F**). The survival of mice was monitored during 14 dpi (**G**). qRT-PCR data (E) are shown as Means ± SD, ****P < 0.0001 by unpaired Student’s* t* test

Furthermore, we assayed the in vivo anti-viral effect of the R3P1-E4 antibody in a mouse model based on a SARS-CoV-2 strain MASCp36 [28]. Upon intranasal infection with 30 PFU of MASCp36, all mice treated with IgG control showed robust viral replication in the lung and trachea at 3 days post infection (dpi), however intraperitoneal administration with 25 mg/kg R3P1-E4 antibody significantly reduced viral RNA loads in both lung and trachea (Fig. 3A, B). The RNA scope and H&E staining assay further showed the strong inhibition of R3P1-E4 antibody on viral RNA deposited in the lung tissues of infected mice (Fig. 3C, D) related lung damage (Fig. 3E). In order to determine the efficacy of R3P1-E4 antibody on rescuing the lethal phenotype of SARS-CoV-2 infected mice, we infected mice with the lethal dose of MASCp36 and treated the infected mice with 25 mg/kg R3P1-E4 antibody and IgG control at 2 h and 24 h post infection (Fig. 3F). As shown in Fig. 3G, while about 40% infected mice treated with IgG dies around day 5 post infection, all the infected mice treated with R3P1-E4 antibody survived beyond two weeks. Taken together, these results demonstrated that R3P1-E4 antibody effectively suppressed SARS-CoV-2 infection and replication in vitro and in vivo.

### R3P1-E4 has variable binding and suppressing activity against different SARS-CoV-2 variants

**Fig. 4:**
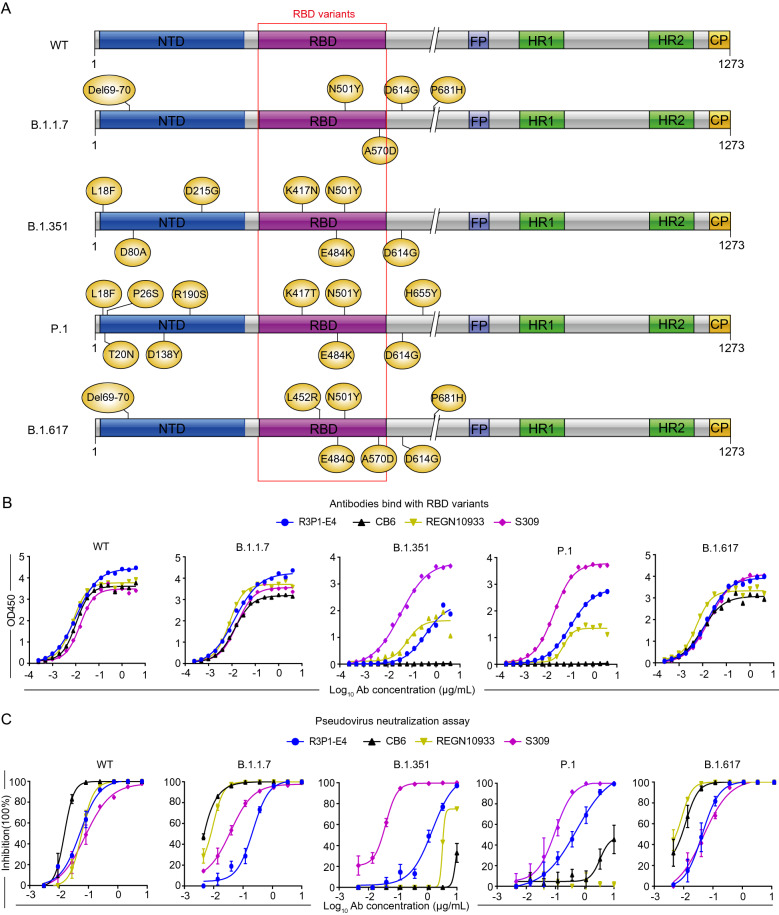
R3P1-E4 suppresses SARS-CoV-2 variants. **A** Sketch map of SARS-CoV-2 pseudoviruses construction. **B** Antibody competition assay were performed to test the abilities of R3P1-E4, CB6, REGN10933 and S309 mAbs to competitively bind with RBD variant proteins against hACE2. **C** The abilities of R3P1-E4, CB6, REGN10933 and S309 mAbs to neutralize SARS-CoV-2 pseudovirus were examined by pseudovirus neutralization assay

The prevalence of SARS-CoV-2 variants arise new challenge of current neutralizing antibody development as some antibodies that effectively inhibited wild type (WT) SARS-CoV-2 partially or totally lost their ability to neutralize SARS-CoV-2 variants. For example, regdanvimab (CT-P59) and, to a smaller extent, etesevimab, showed a reduction in neutralization potency against the B.1.472/B.1.429, whereas bamlanivimab (LY-CoV555) entirely lost its neutralizing activity to B.1.617 due to the central location of L452R in the epitopes recognized by this mAb. In our study, we firstly compared the binding of R3P1-E4, CB6, S309 and REGN10933 mAbs with RBD domains from different SARS-CoV-2 variants including B.1.1.7, B.1.351, P.1 and B.1.617. While R3P1-E4 bound to the RBD proteins of WT and all variants, the binding affinities to B.1.351 and P.1 were reduced (Fig. 4A, B). Interestingly, REGN10933 mAb also had similar reduced binding affinities to P.1 and B.1.351 as R3P1-E4. CB6 showed no binding activities with RBD B.1.351 and P.1, whereas S309 bound to the RBD proteins of WT and all variants at similar affinities. Overall, although R3P1-E4, CB6, S309 and REGN10933 mAbs had similar high binding affinities to the RBD protein of WT SARS-CoV-2, but their binding affinities were differentially affected by mutations in B.1.1.7, B.1.351, P.1 and B.1.617 variants (Fig. 4A, B and Additional file 1: Fig. S2A). In addition to the binding affinities to the RBD proteins, we have also measured the neutralization activities against the infection of pseudoviruse containing the full-length S proteins from WT, B.1.1.7, B.1.351, P.1 and B.1.617 strains. As shown in Fig. 4C, while R3P1-E4, CB6, S309 and REGN10933 mAbs were able to effectively neutralize the WT, B.1.1.7 and B.1.617 pseudoviruses, they all except S309 had significantly reduced their neutralization activities against B.1.351. Interestingly, although both R3P1-E4 and REGN10933 have similar binding affinities to the RBD proteins from P.1, R3P1-E4 had strong neutralization activity against but REGN10933 entirely lost its ability to neutralize the P.1 pseudovirus (Fig. 4C, Additional file 1: Fig. S2B).

### R3P1-E4 antibody binds to the RBD domain through sites overlapping with hACE2

**Fig. 5:**
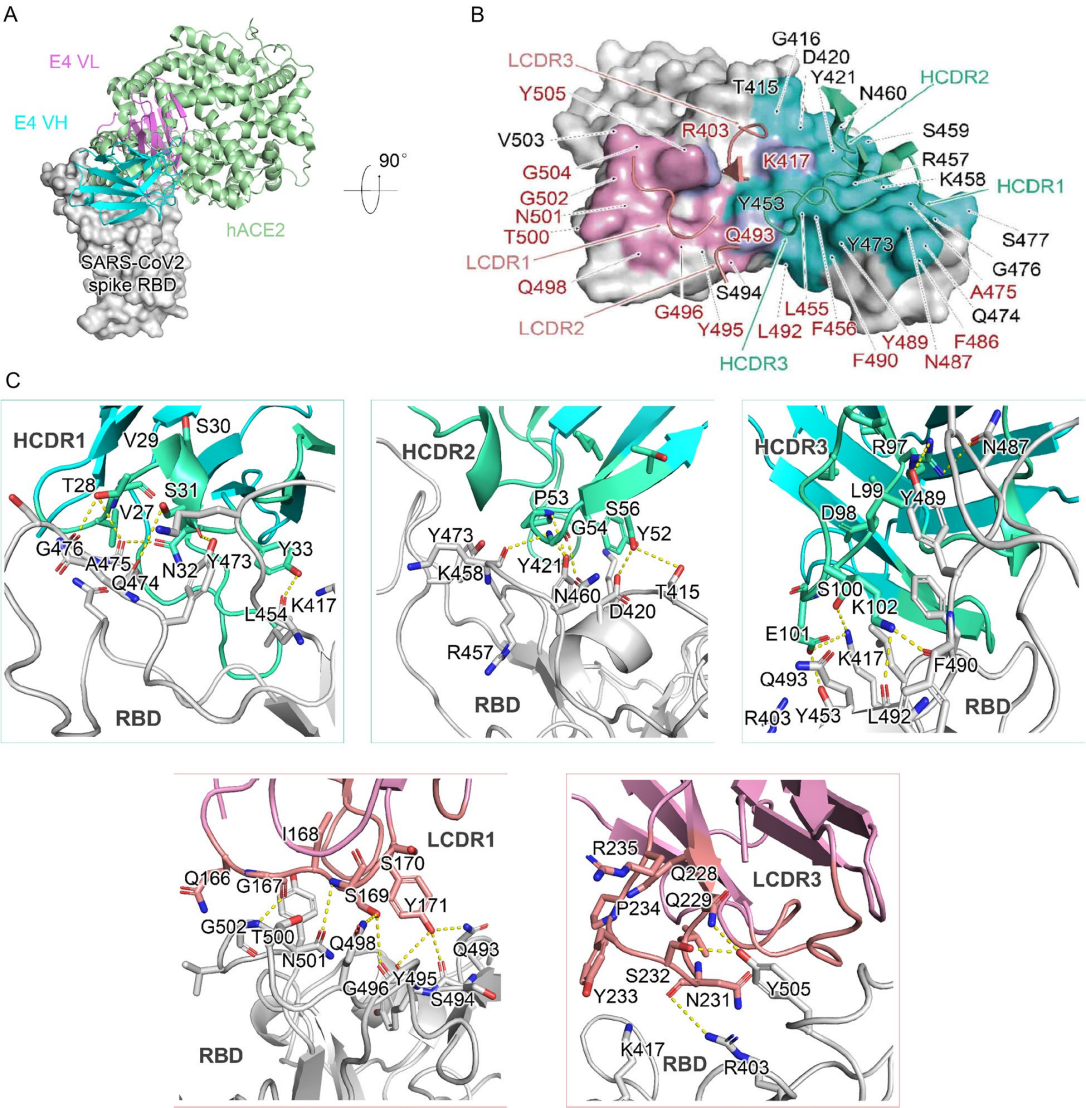
Structural analysis of R3P1-E4 and SARS-CoV-2 RBD complex. **A** The overall complex structure of R3P1-E4-RBD superimposed with the hACE2-RBD complex. The R3P1-E4 heavy chain (colored cyan), light chain (colored violet) and hACE2 (colored pale green) are displayed in cartoon representation. The SARS-CoV-2 RBD is colored in gray and displayed in surface representation. **B** The epitope recognized by R3P1-E4 is shown in surface representation. The CDR loops of heavy chain (HCDR) and light chain (LCDR) are colored in green cyan and salmon, respectively. The epitopes from the heavy chain and light chain are colored in cyan and violet, respectively. The residues of R403, K417 and R493, which contacts with both heavy chain and light chain, is colored in light blue. The identical residues from RBD participating in the binding of R3P1-E4 and hACE2 are labeled in red. These residues are numbered according to SARS-CoV-2 RBD. **C** The detailed interactions between SARS-CoV-2 RBD with HCDRs and LCDRs. The residues are shown in sticks with identical colors to (**B**)

To understand how mutations in different SARS-CoV-2 variants affects the binding and neutralization activities of R3P1-E4, we thought to solve the co-crystal structure of the R3P1-E4 antibody and RBD protein complex. Purified R3P1-E4 scFv protein and the RBD protein were mixed in 1.5:1 molar ratio and their complex was used to generate crystals. The crystal structure of this complex was solved at 2.9 Å with a final R_work_ value of 24.8% (R_free_ = 27.4%) (Additional file 1: Table S1). Both the heavy and light chains of R3P1-E4 bound to the RBD protein through the residues extensively overlapped with the binding sites for hACE2 (Fig. 5A, Additional file 1: Table S2). The topology of the R3P1-E4 antibody in its interaction with the RBD protein was similar as REGN10933, CB6 and B38 antibodies but different from other antibodies such as H014, CR3022 and REGN10987 (Fig. 5B, Additional file 1: Fig. S3A and Table S3). Multiple heavy chain regions including G26-Y33 of HCDR1, I51-T57 of HCDR2 and A96-V107 of HCDR2 and light chain regions including Q166-Y171 of LCDR1 and Q228-R235 of LCDR3 are involved in binding to K417-N487, T415-Y473, R403-Q493, Q493-Y505 and K417-Y505 in the RBD protein, respectively (Fig. 5C, Additional file 1: Fig. S3B). These interaction regions covered all the RBM of SARS-CoV-2 S protein, which is critical for binding to hACE2 during viral entry. Thus, our structural studies suggested that the R3P1-E4 antibody may neutralize SARS-CoV-2 infection through blocking RBM-mediated binding to hACE2. In addition, our structural studies also identified numerous critical amino acid residues involved in interactions between R3P1-E4 antibody and RBD including the proximal contacts of Y33, Y52, S100H, E101 of heavy chain and N231 of light chain with K417 of RBD (Fig. 5C, Additional file 1: Table S2), which may explain why the R3P1-E4 mAb had weaker binding affinity to the RBD proteins from B.1.351 and P.1 strains of SARS-CoV-2 as they contain K417N and K417T mutations, respectively, as compared to that from the WT strain of SARS-CoV-2.

### Improve R3P1-E4 binding and neutralizing activity through antibody engineering

**Fig. 6:**
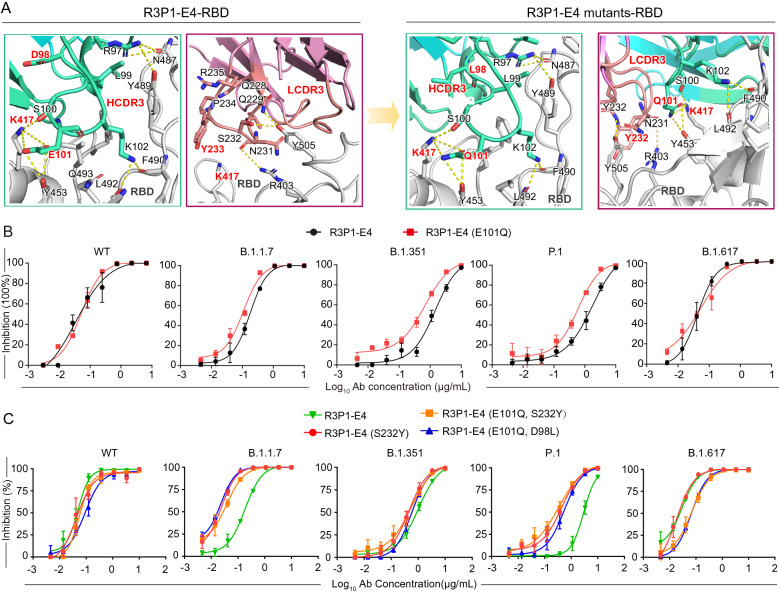
Improve R3P1-E4 binding and neutralizing activity through antibody engineering.** A** The detailed interactions between SARS-CoV-2 RBD with R3P1-E4 and R3P1-E4 mutant mAbs. **B** The abilities of R3P1-E4 and R3P1-E4 (E101Q) mAbs to neutralize SARS-CoV-2 pseudovirus were examined by pseudovirus neutralization assay. **C** The abilities of R3P1-E4 and R3P1-E4 mutant mAbs to neutralize SARS-CoV-2 pseudovirus were examined by pseudovirus neutralization assay

In order to compensate the binding energy loss upon K417N and K417T mutations, we constructed a computational pipe line to screen R3P1-E4 mutants against B.1.351 and P.1 strains of SARS-CoV-2. Briefly, the complementarity determining regions (CDRs) of R3P1-E4 were selected to perform virtual mutations at each position (see “Methods” section) [29, 30]. Then the massive mutants were evaluated by a well-established protein energy scoring function EvoEF2 [31]. The top ranking 50 mutants for both scoring functions were manually inspected to select four mutations (E101Q, S232Y and D98L) for further validation. Our results predicted that the E101Q mutation in the R3P1-E4 heavy chain might increase the binding affinities to the RBD proteins of B.1.351 and P.1 (Fig. 6A). We subsequently generated the E101Q mutation and purified the mutant R3P1-E4 mAb. Our pseudovirus neutralizing assay showed that the E101Q mutation enhanced the ability of R3P1-E4 mAb to neutralize B.1.1.7, B.1.351, and P.1 pseudoviruses (Fig. 6B). Meanwhile, based on the structural analysis of R3P-E4 and RBD, we predicted and generated other R3P1-E4 mutant mAbs and tested the pseudovirus neutralizing effect of these mAbs on SARS-CoV-2 variants. The results showed that among different mutant R3P1-E4 mAbs, the E101Q and D98L mutant was the best to neutralize B.1.1.7 variant whereas the E101Q and S232Y mutant was the best to neutralize P.1 variant (Fig. 6A, C), suggesting the potential of R3P1-E4 mAb remolding to neutralize multiple SARS-CoV-2 variants. Our studies therefore suggested the possible ways to improve the efficacy of R3P1-E4 mAb against different mutant strains of SRAS-CoV-2 viruses through antibody engineering.

## Discussion

Continuously emerging new SARS-CoV-2 variants not only increase the viral transmission but can also escape immune protection by the current vaccines. Although therapeutic antibodies are available to treat COVID-19 patients, they have reduced neutralization activities against the recently evolved SARS-CoV-2 VOCs [32, 33]. Developing strategies to overcome the antibody resistance by emerging VOCs are important to solve the COVID-19 pandemic problems. In this report, we have isolated multiple monoclonal neutralizing Abs from COVID-19 patients with high binding activity to the RBD of SARS-CoV-2 and neutralization activity against SARS-CoV-2 infection (Fig. 2C–E). Among these mAbs, we have demonstrated the strong efficacy of R3P1-E4 antibody in suppressing SARS-CoV-2 infection in mice and rescue its associated lethal phenotype (Fig. 3). Based on the crystal structural analysis, we have identified critical interactions between the R3P1-E4 antibody and the RBD of SARS-CoV-2, which enable to explain K417T/N mutations in certain VOCs are resistant to mAbs and come up with strategy to overcome the resistance by antibody engineering.

We have used the phage display method to isolate human mAbs binding to the RBD of SRAS-CoV-2 directly from COVID-19 patients. This method involved in randomly constructing together with a linker one heavy and one light scFv PCR fragments from COVID-19 patients’ PBMC and expressing them on the phage surface (Fig. 1C). Through purification with the SARS-CoV-2 RBD, we have identified six mAbs with high binding affinity to RBD. Interestingly, all the six mAbs shared the same heavy chain derived from the IGHV3-53 segment with several different light chains from IGKV3-20, IGKV1-9 and IGLV3-21 segments of the human immunoglobulin gene locus. Although the mAbs obtained by this phage display method might not reflect the nature pairs of heavy and light chains in antigen specific B cells, several previously reported mAbs including P4A1, CC12.1, CC12.3 and B38 also contained the same IGHV3-53 segment paired with either IGKV3-20, IGKV1-9 or IGLV3-21 segments, suggesting these heavy and light chain segments are frequently used in COVID-19 patients in response to SARS-CoV-2 infection [3, 34, 35]. We have further shown with the RBD, pseudovirus and authentic SARS-CoV-2 neutralization assays that all our six mAbs had strong efficacy to neutralize the RBD-ACE2 interaction and SARS-CoV-2 infection (Fig. 2C–E). More importantly, we have demonstrated that administration one of these mAbs R3P1-E4 effectively suppressed viral replication and inflammatory response in lung and trachea tissues as well as rescued the lethal challenged in mice infected with a mouse adoptive strain of SARS-CoV-2 (Fig. 3).

Among numerous SARS-CoV-2 VOCs, B.1.351 and P.1 variants are most resistance to antibody neutralization [36]. In addition to the E484K mutation, which has been shown to be responsible for the resistance of many isolated mAbs [37], our studies have suggested that K417N mutation in B.1.351 variant and K417T mutation in P.1 variant are mainly responsible for the decreased neutralization activities of our mAbs and other previously reported mAbs such as CB6 and REGN10933 [38, 39]. Our crystal structural analysis on the R3P1-E4 scFv and RBD protein complex showed that both the heavy and light chain of R3P1-E4 directly interacted with the RBM of SARS-CoV-2 S protein through the residues extensively overlapped with the binding sites for hACE2, suggesting R3P1-E4 neutralizes SARS-CoV-2 infection through competition with hACE2 binding to RBM of the SARS-CoV-2 S protein. Furthermore, we found close contact interactions of K417 of RBD with multiple residues of R3P1-E4 antibody including the Y33, Y52, S100 and E101 of heavy chain and N231 of light chain. These interactions may explain why B.1.351 and P.1 variants, which respectively carry K417N and K417T mutations, were selectively more resistant to R3P1-E4 antibody as compared to WT, B.1.1.7 and B.1.617 strains. More importantly, by introducing E101Q mutation in the heavy chain, we found the engineered R3P1-E4 antibody had improved binding and neutralization activity to both B.1.351 and P.1 variants.

COVID-19 pandemic continues to challenge the world as numerous new variants of SARS-CoV-2 evolve. Many VOCs not only are more infectious but also gain the ability to escape from antibody protection. Our studies together with many others have provided evidence on why certain critical mutations such as E484K, K417N and K417T in the VOCs enable SARS-CoV-2 more likely to break through current vaccines and to become more resistant to antibody treatment. We have also explored the possibility to enhance the binding and neutralization activity of mAbs against VOCs by structure based antibody engineering. Further studies are necessary to determine if vaccination with RBD carrying these critical antibody escaping mutations or treatment with engineered antibodies would significantly improve the protection and therapy of COVID-19 associated with VOCs.

## Methods

### Viruses, cells and antibodies

SARS-CoV-2 original strain (Txid: 2697049) and mouse adapted strain, MASCp36, were used in this study [28]. WT and variant SARS-CoV-2 pseudovirus with *luciferase* coding sequence were constructed in our lab. hACE2 stable expressing HEK293T (hACE2-293T) cell line was established by infected with lentivirus containing hACE2 coding sequence and selected by FACS gating on GFP positive cells. HEK293T and Huh7.5 cell lines were purchased from American Type Culture Collection and cultured in Dulbecco’s modified Eagle’s medium (DMEM) (37 °C, 5% CO_2_) supplemented with 10% fetal bovine serum (FBS), 100 U/mL penicillin, and 50 μg/mL streptomycin. For Huh7.5 cell line, additional 1 × MEM non-essential Amino Acids (MEM NEAA, Gibco) was supplemented. CB6, REGN10933 and S309 mAbs were expressed and purified by AtaGenix Laboratories (Wuhan) Co., Ltd.

### Mouse experiments and ethics statement

Balb/c mice were purchased from the Vital River Laboratory and experimental involving infectious SARS-CoV-2 were performed in biosafety level-3 (BSL-3) facilities. The in vivo SARS-CoV-2 inhibition efficacy of R3P1-E4 was assessed using two well-established SARS-CoV-2 infection mouse models. Firstly, we tested the protection efficacy of R3P1-E4 in mouse model based on a SARS-CoV-2 mouse adapted strain MASCp36 [28]. Briefly, a group of 8-month-old male Balb/c mice were intraperitoneally administrated with R3P1-E4 (25 mg/kg) (n = 5 mice) or IgG control (n = 5 mice) before and after challenge with 30 PFU of MASCp36 via intranasal route. All mice were monitored daily for morbidity and mortality. The lung and trachea tissues of mice were collected at 3dpi for viral RNA loads assay, the lung tissues were also tested by RNA in situ hybridization (ISH) assay and H&E staining.

Then, we tested the therapeutic efficacy of R3P1-E4 in a newly established mouse model based on the SARS-CoV-2 mouse lethal strain MASCp36. Briefly, the 8-month-old male BALB/c mice were intravenous administrated with R3P1-E4 (25 mg/kg) at 1 h and 24 h after challenge with 600 PFU of MASCp36 via intranasal route. All mice were monitored daily for morbidity and mortality until 14 dpi.

### SARS-CoV-2 neutralization assay

The SARS-CoV-2 pseudovirus and authentic virus neutralization ability of mAbs were determined in hACE2-293T cells via *luciferase* reporter or quantitative reverse transcription-PCR (qRT-PCR) assay. Briefly, serial diluted mAbs were mixed with SARS-CoV-2 pseudovirus or authentic virus and incubated at 37 °C for 2 h. The mixture was added to hACE2-293T cells and the cells were subsequently incubated for 24 h. Then, *luciferase* reporter assay was performed to determine the SARS-CoV-2 pseudovirus quantity, and qRT-PCR assay of culture supernatants were performed to determine the genome copies of SARS-CoV-2 authentic virus.

### COVID-19 scFv phage display library construction, biopanning and scFv purification

The method of COVID-19 patients’ scFv phage display library construction, biopanning and scFv purification was described in our previous work [27]. Briefly, the VH and VL gene fragments were PCR amplified using the mixed cDNA from 15 COVID-19 patients’ PBMC as template. The amplified gene fragments were ligated into the pATA-scFv-2 vector which including the M13 Gene III, scFv linker (GGGGSGGGGSGGGGSGGGGAS), multiple restriction sites, Lac promoter, Lac operator and pel B signal peptide. The ligated DNA mixture was electroporated into *E.coli* TG1 cells. Transformed TG1 clones were collected and used to amplify the library phages. The measured capacity of COVID-19-pATA-scFv phage display library is 8.7 × 10^9^ CFU. Specific phages against RBD protein from COVID-19-pATA-scFv phage display library were affinity-enriched by 4 rounds biopanning and unique positive antibodies were obtained by validated ELISA and sequencing analysis.

Transformed TG1 cells were cultured and the supernatant was got by ultracentrifugation, mixed with Ni-TED beads (Roche) and rocked at 4 °C for 30 min. Washed the beads with PBS and verified the purified protein by SDS-PAGE. Cut the target scFv fraction and dialysis against PBS (pH = 7.4). Then, 1% (v/v) Triton X-114 were added to the protein sample. After 30 min stirring at 4 °C, the mixture was incubated at 37 °C water bath for 10 min and centrifuged (2000 *g*, 10 min) at 25 °C. The purified scFv was concentrated from the upper aqueous and stored at −20 °C.

### Surface plasmon resonance assay

The binding of mAbs to RBD protein under laminar flow was analyzed by surface plasmon resonance (SPR) using a BIAcore T200 system (GE Healthcare). The surface of a carboxymethylated dextran (CM5) sensor chip (GE Healthcare) was activated with 0.4 M 1-ethyl-3-(3-dimethylaminopropyl) carbodiimide (ThermoFisher Scientific) and 0.1 M N-hydroxysuccinimide (ThermoFisher Scientific). mAbs was immobilized by amine coupling to one flow cell. All free reactive surface groups were blocked using 1 M ethanolamine. Different concentrations (0–64 nM) of RBD protein in HBS buffer containning 0.005% Tween-20 were injected over the flow cells at 30 μL/min (contact time, 2 min). After each injection, any bound protein was stripped with 10 mM glycine (15 s). Data analysis was performed using the BIAcore T200 evaluation software 3.1 (GE Healthcare). The K_D_ values were calculated and additional lines parallel to the y-axis were added to the figures to mark the location of the K_D_ value.

### Enzyme linked immunosorbent assay

Firstly, the plates were coated with WT or mutant RBD proteins. After washing three times, the plates were blocked with 100 μL/well blocking solution at 37 °C for 1 h. Then the plates were incubated with 100 μL/well indicated antibodies at 4 °C for overnight. After washing three times, the plates were incubated with 100 μL/well HRP-anti-human IgG secondary antibody at 37 °C for 1 h. After washing five times, 100 μL/well TMB were added into the plates and incubated for 5–10 min with light protection. Then the reaction was terminated by adding 50 μL/well 2 M H_2_SO_4_. The absorbance of each well was measure at 450 nm with SpectraMax i3 (Molecular Devices) plate reader.

### Antibody competition with hACE2 receptor

The competitively binding affinity of antibodies to RBD protein was analyzed by ELISA kit (Genscript) according to the manufacturer's instruction. The antibody abilities to competitively binding with S against ACE2 was calculated using OD_450_ of experimental group/OD_450_ of background group.

### Histopathological analysis

Mouse tissues were excised and fixed with 10% neutral buffered formalin, dehydrated and embedded in paraffin. Embedded tissue was sectioned into 4 μm thickness longitudinal sections. Tissue section were stained with hematoxylin and eosin (H&E) according to standard procedures for examination by light microscopy. The lung damage under the light microscopy was assessed by the degeneration of alveolar epithelial cells, the expansion of parenchymal wall, edema, hemorrhage, and inflammatory cells infiltration.

### RNA ISH assay

SARS-CoV-2 genome RNA ISH assay was performed with RNAscope® 2.5 HD Reagent Kit (Advanced Cell Diagnostics) according to the manufacturer’s instruction. Briefly, formalin-fixed and paraffin-embedded tissue sections of 5 μm were deparaffinized by incubation for 60 min at 60 °C. Then, the tissue sections were treated with hydrogen peroxide at room temperature (RT) for 10 min to quench endogenous peroxidases. Tissue sections were then boiled for 15 min in RNAscope Target Retrieval Reagents and incubated for 30 min in RNAscope Protease Plus before probe hybridization. Tissue sections were counterstained with Gill’s hematoxylin and visualized with standard bright-field microscopy. Original magnification was 40× .

### Quantitative reverse transcription-PCR

SARS-CoV-2 viral RNA from cell supernatant was extracted using QIAamp Viral RNA Mini Kit (Qiagen, Cat No. 52904) and viral RNA from mouse tissue samples were extracted by using TRIzol reagent according to the manufacturer’s instruction. SARS-CoV-2 RNA copies in the samples were measured by qRT-PCR using One Step PrimeScript RT-PCR Kit (Takara, Japan). The primers and probe used in qRT-PCR assay were as follows: CoV-F3 (5′-TCCTGGTGATTCTTCAGGT-3′); CoV-R3 (5′-TCTGAGAGAGGGTCAAGTGC-3′); and CoV-P3 (5′-FAM-AGCTGCAGCACCAGCTGTCCA-BHQ1-3′).

### RBD protein expression and purification

The codon optimized cDNA of SARS-CoV-2 RBD (residues 335–530) was synthesized. The SARS-CoV-2 RBD with a C-terminal 8 × His tag for purification was cloned into pAcgp67 vector, and expressed using the Bac-to-Bac baculovirus system. The construction was transfected into DH5α component cells, and the extracted bacmid was then transfected into Sf9 cells using Cellfectin II Reagent (Invitrogen). Amplified the low-titer viruses to generate high-titer virus stock. The viruses and Endo H, Kifunensine were co-infected Hi5 cells at a density of 2 × 10^6^ cells/mL. The supernatant of cell culture containing glycosylated RBD was harvested 72 h post infection, concentrated and RBD was captured by Ni–NTA resin (GE Healthcare). The resin was washed with 30 mL washing buffer (25 mM Tris, 150 mM NaCl, 40 mM imidazole, pH = 7.5) for five times, the target protein was eluted with elution buffer (25 mM Tris, 150 mM NaCl, 500 mM imidazole, pH = 7.5). RBD protein was purified on a Superdex S75 (GE Healthcare) column, and the purity of the final purified recombinant protein was analyzed by SDS-PAGE gel. Fractions from the single major peak were pooled and concentrated to 15 mg/mL.

### Crystallization

The SARS-CoV-2 RBD protein and R3P1-E4 Fab fragment were mixed at a molar ratio of 1.5:1. The mixture was incubated at 4 °C for 1 h, and purified by Superdex S75 (GE Healthcare). Then, 7 and 10 mg/mL of RBD/Fab proteins were used for crystal screening by vapor-diffusion sitting-drop method at 16 °C, including the Index, Crystal Screen, PEG/Ion, SaltRX from Hampton Research, and wizard I-IV from Emerald BioSystems. The rode-like crystal appeared after 2 days at the mother liquid (20% w/v PEG3350, 0.2 M potassium citrate tribasic). Further optimization was performed with additive and hanging-drop vapor-diffusion method, the final optimized diffraction crystals at the mother liquid by the hanging-drop vapor-diffusions method. Crystals were dehydrated and cryo-protected in 4 M Sodium formate solution and cooled in a dry nitrogen stream at 100 K for X-ray data collection.

### X-ray data collection, processing and structure determination

Diffraction data were collected at Shanghai Synchrotron Radiation Facility BL17U1 (wavelength, 0.979155 Å) at 100 K. All data sets were processed using the HKL3000 package [40]. Structures were constructed using PHASER with the SARS-CoV-2 RBD structure (PDB ID:6M0J) and the structure of the Fab fragment available in the PDB with the highest sequence identities by molecular replacement [2, 41]. The initial model was built into the modified experimental electron density using COOT (Version 0.9.4) and further refined in PHENIX (Version 1.19) [42, 43]. Model geometry was verified using the program MolProbity. Structural figures were drawn using PyMOL (Version 1.8) (http://www.pymol.org). Epitope and paratope residues, as well as their interactions, were identified by accessing PISA (http://www.ebi.ac.uk/pdbe/prot_int/pistart.html) at the European Bioinformatics Institute.

### Antibody engineering

Antibody virtual mutations were performed over the CDRs regions of R3P1-E4, which were identified by an antibody numbering tool AbRSA [29]. Residues at the CDRs were enumerated to substitute with 19 types of amino acids in the virtual screening. For each mutant, the 3D structures were constructed with a side-chain modelling method CIS-RR [30], and further optimized using energy minimization to remove the atomic clashes [44]. The predicted mutant structures complexed with RBD were then go through stability as well as affinity evaluation with a recent developed protein energy function EvoEF2. The mutants with increment in stabilities and affinities were reserved for intense investigation. They were ranked in terms of inter van der Waals, electrostatic and desolvation energy. Moreover, the conformational variations between the original R3P1-E4 and the mutants were limited in 1.0 Å to avoid the computational errors. Finally, four mutants were selected for validation.

### Statistics

Statistical analyses were performed with GraphPad Prism 8 software and R Studio version 3.6.3. The continuous variables were presented as mean ± SD. Data with normal distribution were analyzed by one-way ANOVA or unpaired two-tailed Student’s *t* tests, and *P* values were indicated by ns, not significant, ***P < 0.001 and ****P < 0.0001.

## Supplementary Information

**Additional file 1:** **Figure S1.** Germline usage comparison of P4A1, CC12.1, CC12.3 and B38 mAbs.** A** Germline usage comparison of heavy chain and light chain of P4A1, CC12.1, CC12.3 and B38 mAbs. **B** Alignment of the heavy chain and light chain variable domain sequence of P4A1, CC12.1, CC12.3 and B38 mAbs. **Figure S2.** RBD binding and pseudovirus neutralizing activity comparison of R3P1-E4, CB6, S309 and REGN10933 mAbs. **A** The EC_50_ of R3P1-E4, CB6, S309 and REGN10933 mAbs binding with RBD WT and variants. **B** The IC_50_ of SARS-CoV-2 pseudovirus neutralizing activity of R3P1-E4, CB6, S309 and REGN10933 mAbs. **Figure S3.** Structural comparison of the binding mode among R3P1-E4 and several reported RBD-specific neutralizing antibodies from various germlines. **A** Superposition of R3P1-E4 (deep teal, PDB: 7VMU), B38 (orange, PDB: 7BZ5), CB6 (deep purple, PDB: 7C01), H014 (green, PDB: 7CAH), CR3022 (blue, PDB: 6ZH9), REGN10987 (magenta, PDB: 6XDG), REGN10933 (hot pink, PDB: 6XDG), to SARS-CoV-2 spike glycoprotein RBD (gray). **B** Surface representation of several Spike RBD mutations isolated from clinic. The SARS-CoV-2 RBD is colored in gray and displayed in surface representation. The epitope of R3P1-E4 heavy chain (cyan), light chain (pink), residue K417 (light blue) are displayed and colored as Figure 5. The clinic mutations L452, G476, S477, T478, E484, F490, S494 and N501Y, which located at the edge of the R3P1-E4 epitope are colored in lime green. The clinic mutations N354, D364, V367, R408, W436, N439 and v483, which are adjacent to the epitope residues or on the opposite side of the R3P1-E4 epitope, are colored in purple blue. **Table S1.** Data collection and refinement statistics for R3P1-E4-RBD complex. **Table S2.** Residues contributed to interaction between R3P1-E4/SARS-CoV-2 RBD. **Table S3.** PISA analysis of interaction between R3P1-E4/SARS-CoV-2-RBD.

## Data Availability

Crystallographic coordinates of R3P1-E4-RBD complex deposited into the Protein Data Bank with PDB code 7VMU. Further information and requests for resource and regents should be directed to the corresponding authors. All requests for raw data, analyzed data and materials will be promptly reviewed by the corresponding author to verify if the request is subject to any intellectual property or confidentially obligations. Any data and materials that can be shared will be released via a Materials Transfer Agreement. Source data have been provided with this paper for Figs. 1–6 as well as Additional file 1.

## Abbreviations

SARS-CoV-2: Severe acute respiratory syndrome coronavirus
COVID-19: Coronavirus disease-2019
mAbs: Monoclonal antibodies
RBD: Receptor binding domain
RBM: Receptor binding motif
NTD: N-terminal domain
S: Spike
ACE2: Angiotensin converting enzyme 2
SPR: Surface plasmon resonance
