# Supplementary material for: Antibody engineering improves neutralization activity against K417 spike mutant SARS-CoV-2 variants
Source: Cell Biosci. 2022 May 17;12:63. doi: 10.1186/s13578-022-00794-7 (PMC9113379; doi:10.1186/s13578-022-00794-7)
Supplement: Supplementary file 1 — Additional file 1: Figure S1. Germline usage comparison of P4A1, CC12.1, CC12.3 and B38 mAbs. A Germline usage comparison of heavy chain and light chain of P4A1, CC12.1, CC12.3 and B38 mAbs. B Alignment of the heavy chain and light chain variable domain sequence of P4A1, CC12.1, CC12.3 and B38 mAbs. Figure S2. RBD binding and pseudovirus neutralizing activity comparison of R3P1-E4, CB6, S309 and REGN10933 mAbs. A The EC50 of R3P1-E4, CB6, S309 and REGN10933 mAbs binding with RBD WT and variants. B The IC50 of SARS-CoV-2 pseudovirus neutralizing activity of R3P1-E4, CB6, S309 and REGN10933 mAbs. Figure S3. Structural comparison of the binding mode among R3P1-E4 and several reported RBD-specific neutralizing antibodies from various germlines. A Superposition of R3P1-E4 (deep teal, PDB: 7VMU), B38 (orange, PDB: 7BZ5), CB6 (deep purple, PDB: 7C01), H014 (green, PDB: 7CAH), CR3022 (blue, PDB: 6ZH9), REGN10987 (magenta, PDB: 6XDG), REGN10933 (hot pink, PDB: 6XDG), to SARS-CoV-2 spike glycoprotein RBD (gray). B Surface representation of several Spike RBD mutations isolated from clinic. The SARS-CoV-2 RBD is colored in gray and displayed in surface representation. The epitope of R3P1-E4 heavy chain (cyan), light chain (pink), residue K417 (light blue) are displayed and colored as Figure 5. The clinic mutations L452, G476, S477, T478, E484, F490, S494 and N501Y, which located at the edge of the R3P1-E4 epitope are colored in lime green. The clinic mutations N354, D364, V367, R408, W436, N439 and v483, which are adjacent to the epitope residues or on the opposite side of the R3P1-E4 epitope, are colored in purple blue. Table S1. Data collection and refinement statistics for R3P1-E4-RBD complex. Table S2. Residues contributed to interaction between R3P1-E4/SARS-CoV-2 RBD. Table S3. PISA analysis of interaction between R3P1-E4/SARS-CoV-2-RBD. [file 13578_2022_794_MOESM1_ESM.docx]

Supplementary Materials for

**Antibody engineering improves neutralization activity against K417 spike mutant SARS-CoV-2 variants**

Lili Li^1, 2#^, Meiling Gao^1, 2#^, Peng Jiao^3#^, Shulong Zu^1, 2#^, Yong-qiang Deng^4#^, Dingyi Wan^5#^, Yang Cao^6#^, Jing Duan^5^, Saba R Aliyari^7^, Jie Li^8^, Yueyue Shi^1, 2^, Zihe Rao^3, 9*^, Cheng-feng Qin^4*^, Yu Guo^3, 9*^, Genhong Cheng^7*^, Heng Yang^1,2*^

^1^Institute of Systems Medicine, Chinese Academy of Medical Science & Peking Union College, Beijing 100005, China

^2^Suzhou Institute of Systems Medicine, Suzhou 215123, China

^3^State Key Laboratory of Medicinal Chemical Biology and College of Life Sciences, Nankai University, Tianjin 300071, China

^4^Department of Virology, State Key Laboratory of Pathogen and Biosecurity, Beijing Institute of Microbiology and Epidemiology, AMMS, Beijing 100071, China

^5^AtaGenix Laboratories (Wuhan) Co., Ltd., Wuhan 430075, China

^6^Center of Growth, Metabolism and Aging, Key Laboratory of Bio-Resource and Eco-Environment of Ministry of Education, College of Life Sciences, Sichuan University, Chengdu 610065, China

^7^Department of Microbiology, Immunology & Molecular Genetics, University of California, Los Angeles, Los Angeles, CA 90095, USA

^8^Department of Laboratory Medicine, Taihe Hospital, Hubei University of Medicine, Shiyan 442000, China

^9^Guangzhou Laboratory, B1, Standard Property Unite 4, Guangzhou international bio-island, Guangzhou 510320, China

**^#^**L. L., M. G., P. J., S. Z., Y-Q. D., D. W. and Y. C. share the co-first authorship.

**^*^**H. Y., G. C., Y. G., C-F. Q. and Z. R. are co-corresponding authors.

Lead contact: Heng Yang [yhmyt@hotmail.com](mailto:yhmyt@hotmail.com)

**This file includes:**

Fig. S1-S3

Table S1-S3


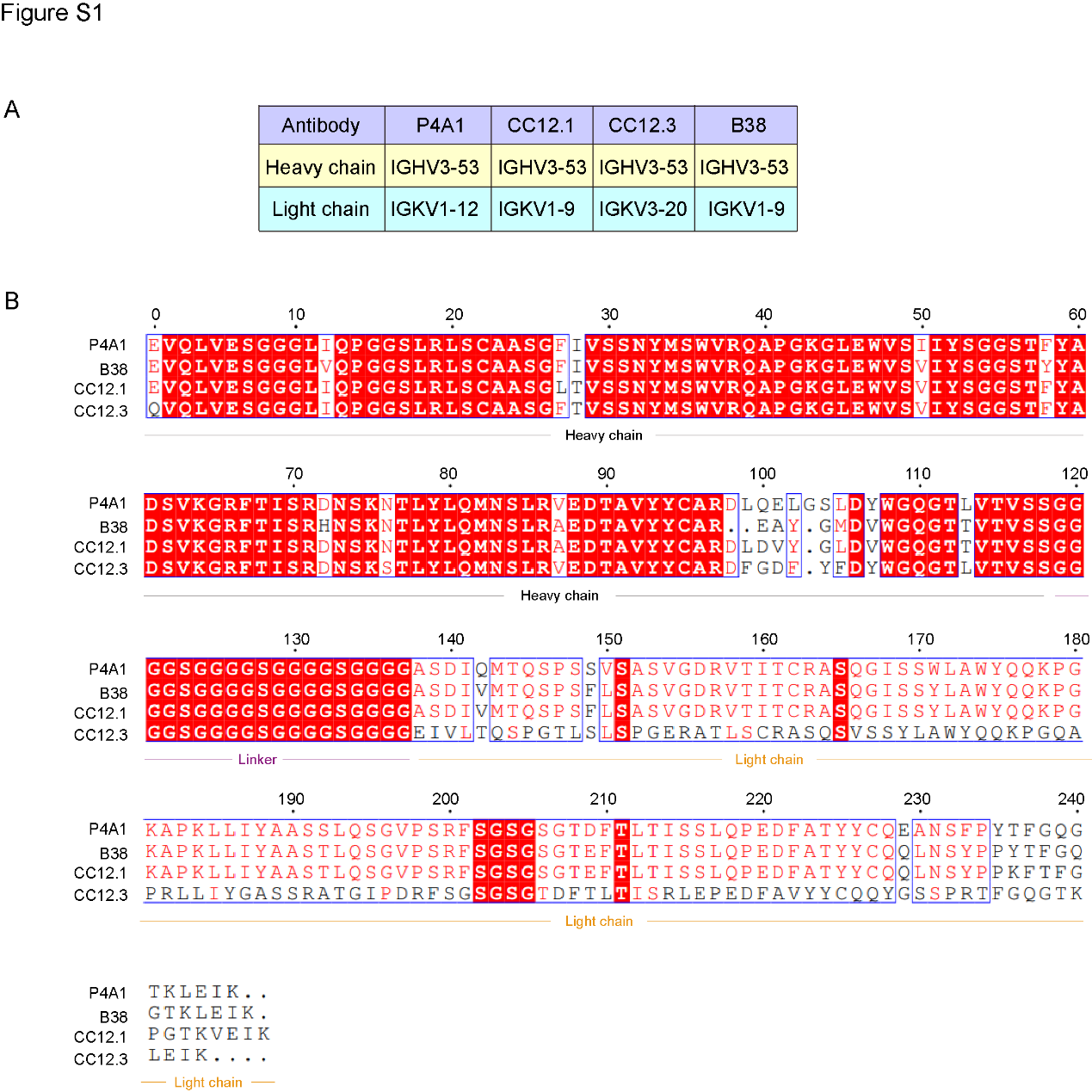


**Fig. S1. Germline usage comparison of P4A1, CC12.1, CC12.3 and B38 mAbs.** **A** Germline usage comparison of heavy chain and light chain of P4A1, CC12.1, CC12.3 and B38 mAbs. **B** Alignment of the heavy chain and light chain variable domain sequence of P4A1, CC12.1, CC12.3 and B38 mAbs.


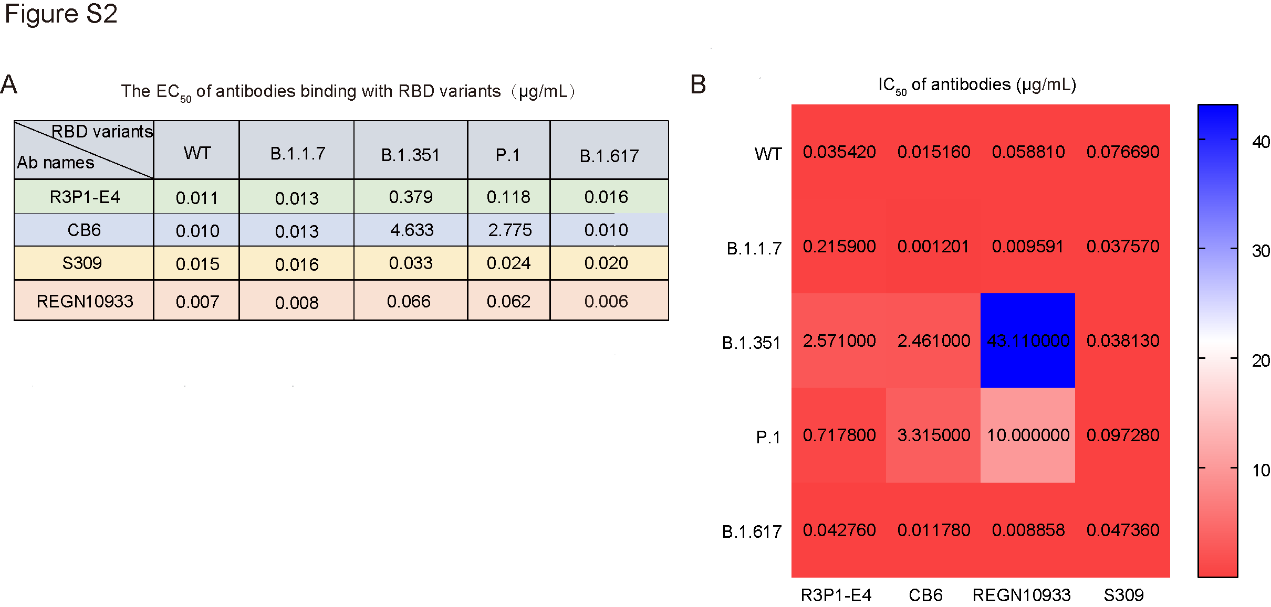


**Fig. S2. RBD binding and pseudovirus neutralizing activity comparison of** **R3P1-E4, CB6, S309 and REGN10933 mAbs.** **A** The EC_50_ of R3P1-E4, CB6, S309 and REGN10933 mAbs binding with RBD WT and variants. **B** The IC_50_ of SARS-CoV-2 pseudovirus neutralizing activity of R3P1-E4, CB6, S309 and REGN10933 mAbs.


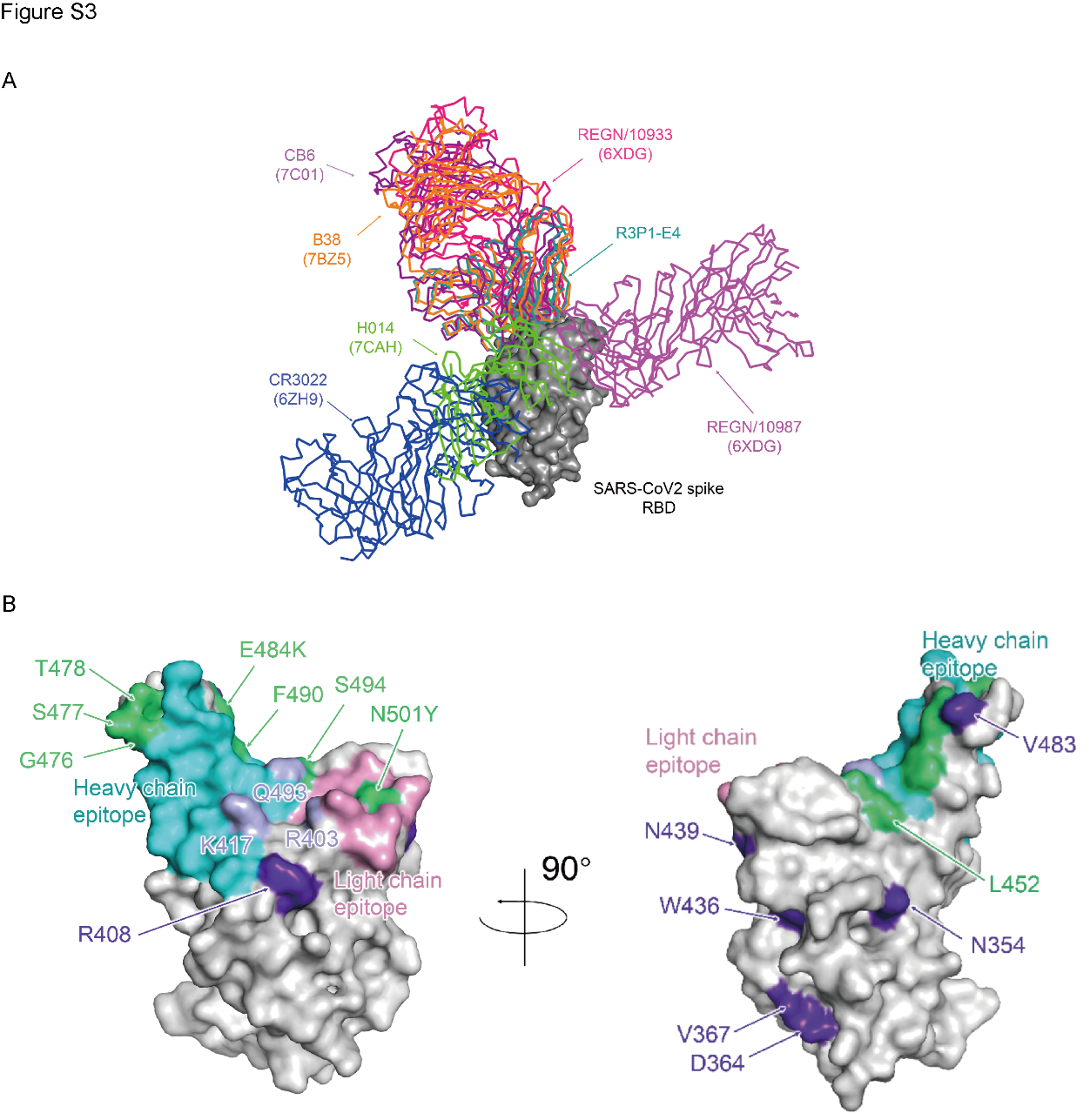


**Fig. S3. Structural comparison of the binding mode among R3P1-E4 and several reported RBD-specific neutralizing antibodies from various germlines.** **A** Superposition of R3P1-E4 (deep teal, PDB: 7VMU), B38 (orange, PDB: 7BZ5), CB6 (deep purple, PDB: 7C01), H014 (green, PDB: 7CAH), CR3022 (blue, PDB: 6ZH9), REGN10987 (magenta, PDB: 6XDG), REGN10933 (hot pink, PDB: 6XDG), to SARS-CoV-2 spike glycoprotein RBD (gray). **B** Surface representation of several Spike RBD mutations isolated from clinic. The SARS-CoV-2 RBD is colored in gray and displayed in surface representation. The epitope of R3P1-E4 heavy chain (cyan), light chain (pink), residue K417 (light blue) are displayed and colored as Figure 2. The clinic mutations L452, G476, S477, T478, E484, F490, S494, and N501Y, which located at the edge of the R3P1-E4 epitope are colored in lime green. The clinic mutations N354, D364, V367, R408, W436, N439 and v483, which are adjacent to the epitope residues or on the opposite side of the R3P1-E4 epitope, are colored in purple blue.

**Table S1. Data collection and refinement statistics for R3P1-E4-RBD complex.**

| **Parameters** |  |  | |
| --- | --- | --- | --- |
| **Data collection statistics** |  |  | |
| a (Å) | 97.02 | |  |
| b (Å) | 97.02 | |  |
| c (Å) | 93.24 | |  |
| α, β, γ (°) | 90, 90, 90 | |  |
| Space group | *P4_3_22* | |  |
| Wavelength used(Å) | 0.9792 | |  |
| Resolution(Å) | 50.00-2.90 (2.95-2.90) ^c^ | |  |
| No. of unique reflections | 9509(751) | |  |
| Completeness (%) | 95.2(96.7) | |  |
| Average I/σ (I) | 13.6(1.2) | |  |
| R_merge_^a^ (%) | 15.7(131.5) | |  |
| No. of reflections used (σ(F) > 0) | 9489 (751) | |  |
| **Refinement statistics** | | |  |
| R_work_^b^ (%) | 24.77 (35.65) | |  |
| R_free_^b^ (%) | 27.40 (39.99) | |  |
| r.m.s.d. bond distance(Å) | 0.011 | |  |
| r.m.s.d. bond angle(º) | 1.36 | |  |
| Average B-factor(Å^2^) | 49.33 | |  |
| Rotamer outliers (%) | 6.59 | |  |
| **Ramachandran plot** | | |  |
| Res. in favored regions (%) | 90.98 | |  |
| Res. in generously allowed region (%) | 8.76 | |  |
| Res. in disallowed region (%) | 0.26 | |  |

^a^ *R_merge_* = Σ_h_Σ_l_ | I_ih_−<I_h_> |/Σ_h_Σ_I_ <I_h_>, where <I_h_> is the mean of the observations I_ih_ of reflection h.

^b^ *R_work_* = Σ(||F_p_(obs)|−|F_p_(calc)||)/ Σ|F_p_(obs)|; *R_free_* is an R factor for a pre-selected subset (5%) of reflections that was not included in refinement.

^c^ Numbers in parentheses are corresponding values for the highest resolution shell.

**Table S2. Residues contributed to interaction between R3P1-E4/SARS-CoV-2 RBD.**

| **SARS-CoV-2 RBD** | **Distance (Å)** | **(Å) P4A1 Antibody** |
| --- | --- | --- |
| Hydrogen Bonds | | |
| LEU 455 [O] | 2.7 | VH:TYR 33(OH) |
| TYR 473 [OH] | 2.8 | VH:SER 31(O) |
| GLN 474 [O] | 4.0 | VH:SER 31(OG) |
| ALA 475 [O] | 3.0 | VH:ASN 32 [OD1] |
| ALA 475 [O] | 3.9 | VH:THR 28 [OG1] |
| GLY 476 [O] | 3.1 | VH:THR 28 [OG1] |
| THR 415 [OG1] | 4.0 | VH:SER 56 [OG] |
| ASP 420 [OD2] | 2.8 | VH:SER 56 [OG] |
| TYR 421 [OH] | 3.6 | VH:PRO 53 [N] |
| TYR 421 [OH] | 2.6 | VH:GLY 54 [N] |
| LYS 458 [O] | 3.8 | VH:GLY 54 [N] |
| ASN 460 [OD1] | 3.3 | VH:GLY 54 [O] |
| LYS 417 [NZ] | 2.8 | VH:SER 100 [OG] |
| TYR 453 [OH] | 3.2 | VH:GLU 101 [OE2] |
| ASN 487 [OD1] | 3.5 | VH:ARG 97 [NH1] |
| TYR 489 [OH] | 3.9 | VH:ARG 97 [NH2] |
| PHE 490 [OH] | 2.4 | VH:LYS 102 [NZ] |
| LEU 492 [O] | 3.1 | VH:LYS 102 [NZ] |
| GLN 493 [NE2] | 3.1 | VL:TYR 171 [OH] |
| SER 494 [O] | 2.5 | VL:TYR 171 [OH] |
| TYR 495 [O] | 3.6 | VL:TYR 171 [OH] |
| GLY 496 [O] | 3.5 | VL:SER 169 [OG] |
| GLN 498 [NE2] | 3.5 | VL:SER 169 [OG] |
| ASN 501 [OD1] | 3.4 | VL:SER 169 [N] |
| GLY 502 [N] | 2.6 | VL:GLY 167 [O] |
| ARG 403 [NH2] | 3.9 | VL:ASN 231 [O] |
| TYR 505 [OH] | 2.7 | VL:GLN 229 [NE2] |
| TYR 505 [OH] | 3.2 | VL: SER 232 [OG] |
| Salt Bridge | | |
| LYS 417 [NZ] | 3.8 | VH:GLU 101 [OE2] |

**Table S3. PISA analysis of interaction between R3P1-E4/SARS-CoV-2-RBD**

|  | Total surface area, Å^2^ | | Interaction residues | | Interface area, Å^2^ | ΔiG  (kcal/m) | ΔiG  (P-value) | N_HB_ | N_SB_ | N_DS_ |
| --- | --- | --- | --- | --- | --- | --- | --- | --- | --- | --- |
|  | RBD | Es4 | RBD | E4 |  |  |  |  |  |  |
| E4-VH | 9708 | 5895 | 24 | 19 | 791.6 | 0.9 | 0.761 | 15 | 1 | 0 |
| E4-VL |  | 5539 | 13 | 11 | 385.0 | 1.3 | 0.798 | 8 | 0 | 0 |

HC: Heavy chain; LC: Light Chain; ΔiG: Solvation free energy gain upon formation of the interface; N_HB_: number of potential hydrogen bonds across the interface; N_SB_: number of potential salt bridges across the interface; N_DS_: number of potential disulfide bonds across the interface; CSS: Complexation Significance Score
